# Comparative Evaluation of Microleakage in Conventional and RMGIC Restorations following Conventional and Chemomechanical Caries Removal: An *in vitro* Study

**DOI:** 10.5005/jp-journals-10005-1259

**Published:** 2015-02-09

**Authors:** Chaitanya Pavuluri, Sivakumar Nuvvula, Rekha Lakshmi Kamatham, SVSG Nirmala

**Affiliations:** Senior Lecturer, Department of Pedodontics and Preventive Dentistry, Drs Sudha and Nageswara Rao Siddhartha Institute of Dental Sciences, Vijayawada, Andhra Pradesh, India; Professor and Head, Department of Pedodontics, Narayana Dental College, Nellore Andhra Pradesh, India; Senior Lecturer, Department of Pedodontics and Preventive Dentistry Narayana Dental College, Nellore, Andhra Pradesh, India; Professor, Department of Pedodontics and Preventive Dentistry Narayana Dental College, Nellore, Andhra Pradesh, India

**Keywords:** Carisolv, Microleakage, Chemomechanical caries removal, Resin-modified glass ionomer, Minimally invasive.

## Abstract

**Background:** Conventional caries removal involves use of high-speed handpiece. Current concepts of caries excavation in cavitated lesions consist of manual excavators. Principles of minimal invasive approach indicate the need to excavate only carious tissue.

**Aim:** The aim of this study was to compare the microleakage in conventional and resin modified glass ionomer cement restorations following conventional and chemomechanical caries removal.

**Design:** Hundred class I carious human mandibular first molar s were collected and divided into two groups: I and II (50 each). Each group was further divided into subgroups, i.e. (IA, IB and IIA, IIB). Caries was completely removed using conventional method in group one and chemomechanically in group two. The teeth in group IA, IIA are restored with conventional glass ionomer comment (GIC) and in group IB, IIB restored with resign-modified glass ionomer comments (RMGIC), followed by fnishing and polishing. Subsequently, the specimens were thermocycled and then placed in dye solution. The teeth were sectioned through the restorations and evaluated for microleakage scores using a stereomicroscope. The data were analyzed using Mann-Whitney U-test.

**Results:** Statistical analysis showed no significant difference in microleakage between the conventional GIC and RMGIC following conventional and chemomechanical caries removal method.

**Conclusion:** Carisolv is minimally invasive and an effective alternative treatment for the removal of occlusal caries.

**How to cite this article:** Pavuluri C, Nuvvula S, Kamatham RL, Nirmala SVSG. Comparative Evaluation of Microleakage in Conventional and RMGIC Restorations following Conventional and Chemomechanical Caries Removal: An *in vitro* Study. Int J Clin Pediatr Dent 2014;7(3):172-175.

## INTRODUCTION

Dental caries, one of the most common chronic oral infections, is the largest cause of tooth loss.^1,2^ Conventional caries removal causes excessive loss of tooth structure. Currently, there is an increased effort toward less invasive removal of caries lesions.^3^ Minimally invasive dentistry is intended to preserve as much sound enamel and den-tin as possible during caries removal.^3,4^ Over the years techniques of caries removal include conventional caries removal, air abrasion with aluminum oxide, atraumatic restorative therapy (ART), Lasers and chemomechani-cal caries removal (CMCR).^4,5^ Chemomechanical caries removal, a more minimalistic approach in treating caries has a considerable potential in the treatment of patients with management problems, especially in pediatric dentistry.

Chemomechanical caries removal with Carisolv^®^, caries is dissolved first by chemical means and then is removed by gentle mechanical excavation. Composition of Carisolv^®^ includes sodium hypochlorite and three amino acids namely: lysine, leucine and glutamic acid.^2,6^ It has the advantage of selective removal of severely dem-ineralized dentin and has been presenting good outcomes compared to the conventional system.^1,7,8^

For many decades, amalgam has been the standard restorative material in dentistry. The detrimental environmental effects of mercury have resulted in a considerable reduction of its use in dentistry. The conventional GIC has many attractive features, such as adhesion to tooth structure, a slow release of fuoride that has cariostatic action, good biocompatibility and a shade similar to tooth but the main disadvantages are the low fracture toughness and poor resistance to wear.^9^

The longevity of a restoration depends on good marginal sealing, thereby reducing marginal leakage, which is the precursor of the secondary caries, marginal deterioration, postoperative sensitivity and pulpal pathology.^10^ One of the factors that can infuence microleakage is the method of caries removal. The study of microleakage thus would contribute to a better assessment of both technique of caries removal and also the restorative material used. Therefore, the present study is designed with the aim to evaluate and compare the microleakage of conventional glass ionomer and resin-modified glass ionomer cements in class I restorations on mandibular first permanent molars, following conventional and chemomechanical caries removal methods.

## MATERIALS AND METHODS

Extracted human molar teeth with class I carious lesions were collected, cleaned using ultrasonic scalers and stored in 0.2% thymol solution at room temperature. The collected teeth were divided into two groups: I and II (50 each). Each group was divided into subgroups, i.e. IA, IB and IIA, IIB. Caries was removed using conventional method (Airotor) in group I and chemomechanically in group II. Caries involving a depth of 2 mm or less from the central pit of the tooth toward the dentine were included in the study. Carisolv^®^ is marketed as two separate solutions, to be mixed prior to application on the carious lesion.

Following the manufacturer's instructions,^11^ equal amount of two components were mixed. The mixed gel was then applied to the carious dentin and left for 30 seconds. The softened carious dentin was then removed using a spoon excavator (Malifer, Germany SS). The procedure was repeated until the carisolv^®^ gel no longer looks cloudy and dentin surface felt hard when probed with a sharp dental explorer.

**Fig. 1:**
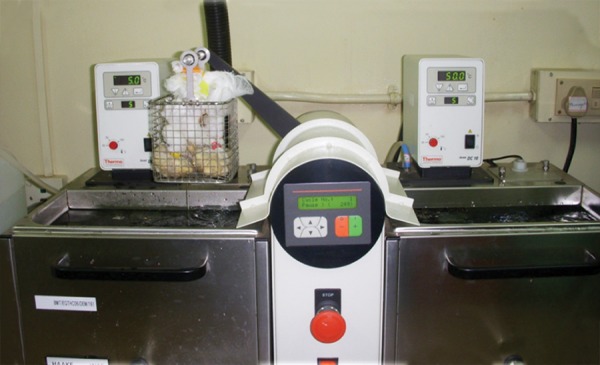
Thermocycling process

In group IA, IIA, the prepared cavities were dried for 5 seconds using oil free air spray and flled with glass iono-mer, Fuji IX™ a nd i n group IB, IIB, the cavities were flled with resin-modified glass ionomer cement (Ketac Nano). Final fnishing of restorations was done. Teeth were then subjected to thermocycling (Dental Thermocycler, Fig. 1) for 250 cycles at a temperature of 5° and 50°C with dwell time of 15 seconds. Each tooth was covered with nail varnish except an area approximately within 2 mm of the periphery of the restoration. The root apices sealed using sticky wax. The specimens were immersed in 0.5% basic fuchsine solution for 24 hours, removed and washed under running water. The teeth were split in buccolin-gually through the restoration using diamond disks and sectioned splits were examined under a stereomicroscope at 18× magnification to determine microleakage scores. Under the stereomicroscope, the depth of the dye penetration was measured and the score which was higher was given as score to that particular tooth.

The following scoring criteria are used:^10^

0― No dye penetration

1― Dye penetration between the restoration and the tooth into enamel only

2― Dye penetration between the restoration and the tooth in the enamel and dentin.

3― Dye penetration between the restoration and the tooth into the pulp chamber.

The scores were tabulated, interpreted and the results were statistically analyzed using Mann-Whitney U- test using SPSS software.

## RESULTS

In 25 teeth restored with conventional GIC (Fuji IX), one tooth showed dye penetration upto enamel, i.e. score 1. Resin-modified glass ionomer cement (Ketac nano) restored teeth showed dye penetration upto dentin with score 2 in 1 tooth and dye penetration upto enamel with score 1 in 2 teeth, following conventional method (airotor) of caries removal. Similarly, 25 teeth restored with conventional GIC (Fuji IX) showed dye penetration upto enamel with score 1 in 2 teeth and in 25 teeth restored with resin-modified glass ionomer cement (Ketac nano) showed dye penetration upto dentin with score 2 in 1 tooth and upto enamel with score of 1 in 3 teeth, following chemomechanical method of caries removal (Table 1).

**Table 1:** **Table 1:** Microleakage pattern in conventional GIC (Fuji IX) and light cured nano ionomer (Ketac nano) restorations following caries removal with conventional and chemomechanical methods (Carisolv^®^)

*Caries removal method*		*Restorative material*		*No. of samples*		*Microleakage scores*								*Mean ± SD*	
						*0*		*1*		*2*		*3*			
Conventional (Airotor)		Fuji IX (IA)		25		24		1		-		-		0.04 ± 0.2	
		Ketac (IB)		25		22		2		1		-		0.16 ± 0.4	
Chemomechanical caries removal (Carisolv^®^)		Fuji IX (IIA)		25		23		2		-		-		0.08 ± 0.2	
		Ketac (IIB)		25		21		3		1		-		0.2 ± 0.5	

## DISCUSSION

Dental care is an integral component of a child's overall healthcare. Different tooth colored restorative materials were used to treat carious teeth and the first of them being the silicate cements till the emergence of GIC and it has emerged as the most frequently used alternative to amalgam.

Glass ionomer cements is considered the only material with self adherence to dental tissue.^12^ The anticariogenic property resulting from fuoride release turned out to be the most attractive aspect of GIC. However, compre -ssive strength of GIC is questionable as is its wear resistance and color stability in posterior teeth. To overcome these, materials that incorporate light curable resin and increased fller content, i.e. resin-modified glass ionomer cements (RMGIC) were developed. Resin-modified glass ionomer cements resulted in the early development of higher bond strength, reduced brittleness, lower moisture sensitivity, reduced solubility and wear resistance and it has antibacterial characteristics.^13^ The integrity of the tooth restoration interface is dependent on several factors, such as polymerization shrinkage at the time of cure, water absorption, and the difference between the linear coefficient of thermal expansion.^5^ The occlusal surfaces of the molars are susceptible surfaces for caries and the occlusal cavities could be easily standardized. Class I restorations were thought to be more practical for microleakage study.^10^ The present *in vitro* study was carried out in class I cavities prepared on extracted permanent mandibular 1st molars.

Carious dentin is composed of two layers – the infected and the affected. Of the two, affected dentin is mineralizable, since the damage is reversible.^14^ Contemporary concepts of caries management deal with preservation of this structure and these concepts are inherited from minimal intervention dentistry. Minimally invasive dentistry is intended to preserve as much sound enamel and dentin as possible during the treatment of carious lesions.^3,4^ Different procedures of caries removal leave distinctly different surface textures and smear layer, thickness of excavated dentin can affect the quality of bonding to dentin and marginal seal.^5^

Chemomechanical excavation is more time consuming than traditional bur excavation but the estimated quantity of tissue removed is significantly lower.^4^ Keeping in view the advantages of Carisolv^®^, it was selected for the present study. The idea of chemomechanical caries removal was developed in 1970s by Goldman, an Endo-dontist, as he was using sodium hypochlorite (NaOCl) in removing organic materials in the root canals. Because of the ability of this chemical to dissolve carious dentin, the idea of removing caries chemically was borne.^15^

The Carisolv^®^ system was developed by the Swedish Medi Team. According to Munshi AK et al, Carisolv^®^ proved to be an effective, atraumatic treatment modality with potential interest for use in clinical pediatric dentistry.^16^ Rafque et al^17^ carried out a clinical trial of combined use of air abrasion/Carisolv^®^, concluded that air abrasion/Carisolv^®^ treatment was a well accepted and viable alternative to conventional method. It has also been suggested that the system may be useful for nervous patients and those who, for medical reasons should not be given a local anesthetic, e.g. hemophiliacs.^14^

Average time required for complete caries removal is about 9 to 12 minutes and the volume of gel is about 0.2 to 1.0 ml.^18^ In the present study, approximately 0.10 ml of Carisolv^®^ gel is used for caries excavation per tooth. Yip HK et al carried out an *in vitro* study, on permanent and deciduous teeth using chemomechanical caries removal and observed 68.4% complete caries removal in permanent teeth and 81% in deciduous teeth.^14^ Mousavinenasab SM, Jafary^5^ concluded from their study that there were no significant differences in microleakage between conventional and chemomechanical caries removal methods, which supports the results obtained in the present study when both the caries removal methods were evaluated.

Kevin J et al stated that RMGIC cement restorations demonstrated a better success rate than that of the conventional GIC restorations.^19^ In the present study, both methods mai nt a in same stabi lit y and did not significantly infuence the success rates of the restorations. Qvist et al, in their study reported longer survival period of RMGIC material with cavity conditioning than without.^20^

The quality of bonding to dentin could be affected to a greater extent by the mode of caries removal. The chemomechanical caries removal showed more irregular and rougher surfaces with modified smear layer when compared with the conventional rotary preparation, as stated by Elkholany NR et al.^15^ Okida RC, Martins TM, Briso AL (2007)^8^ carried out an *in vitro* study to evaluate and compare the occurrence of marginal leakage in two different bonded restorations using mechanical and chemomechanical (Carisolv^®^) removal of carious tissue. After accomplishment of the restorations, the teeth were thermocycled and exposed to dye and they concluded that the system of carious removal did not infuence the results of microleakage for margins in enamel and dentin/cementum.

Thus, the chemomechanical caries removal technique using Carisolv^®^ can be considered as an effective atrau-matic treatment modality. Carisolv^®^ a virtually painless, noninvasive technique of caries removal appears to be of potential interest for use in clinical pediatric dentistry and acts as an aid in combination with the atraumatic restorative treatment of dental caries in large populations.

## CONCLUSION

The results of this study suggest that Carisolv is an effective alternative treatment for the removal of occlusal caries. It helps to preserve dental tissue, although the clinical time spent is longer than that spent when using high-speed excavation.

Comparatively Carisolv is a better alternative treatment for caries removal.

Carisolv, a minimalistic approach has considerable potential in treating anxious patients.
